# Supplementary material for: Poly (Ethylene Oxide)-Based Block Copolymer Electrolytes Formed via Ligand-Free Iron-Mediated Atom Transfer Radical Polymerization
Source: Polymers (Basel). 2020 Apr 1;12(4):763. doi: 10.3390/polym12040763 (PMC7240491; doi:10.3390/polym12040763)
Supplement: Supplementary file 1 [file polymers-12-00763-s001.pdf]

Supporting Information for

Poly (ethylene oxide)-based block copolymer electrolytes formed via ligand-free iron-mediated atom transfer radical polymerization

Sibo Li[1,2], Mengying Tian[1], Jirong Wang[2], Feipeng Du[1], Liang Li[1]*, Zhigang Xue[2]*

[1] *School of Materials Science and Engineering, Wuhan Institute of Technology, Wuhan 430074, China.*
[2] *Key Laboratory for Material Chemistry of Energy Conversion and Storage, Ministry of Education, School of Chemistry and Chemical Engineering, Huazhong University of Science and Technology, Wuhan 430074, China.*

Correspondence to: Z. Xue (E-mail: zgxue@mail.hust.edu.cn) or L. Li (E-mail: msell08@163.com)

Table of Contents:

Supporting Figures
Figure S1. ^{1}H NMR spectrum of PEO_{2000}-Br.
Figure S2. First-order plots (a) and evolution of M_n and $Đ$ with conversion (b) for the bulk $FeBr_2$-catalyzed MMA polymerization in the presence of PEO_{2000}-Br.
Figure S3. Original GPC data with different monomer conversion related to Figure S2.
Figure S4. TGA of PMMA, PMMA-PEO_{2000} and PPEGMA-PEO_{2000}.
Figure S5. Original ac impedance spectra for calculating the ionic conductivity in Figure 5.

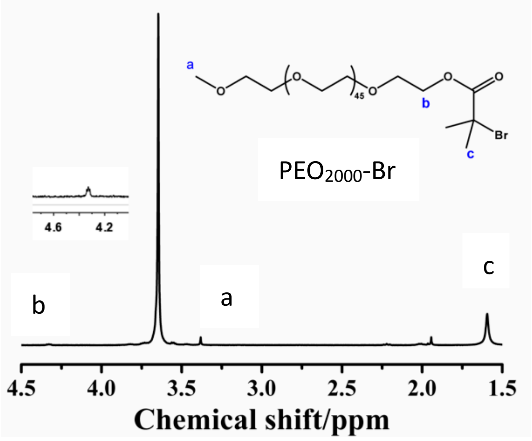

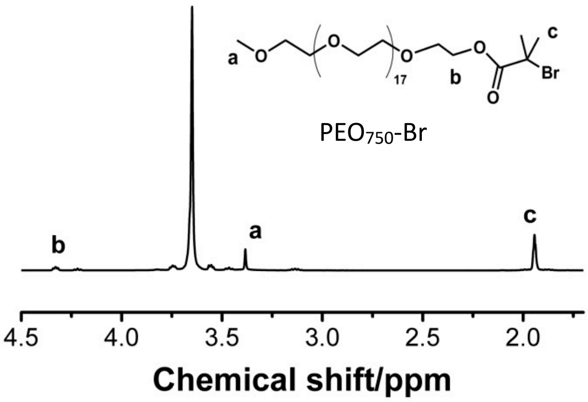

Figure S1. ^{1}H NMR spectrum of PEO_{2000}-Br and PEO_{750}-Br.

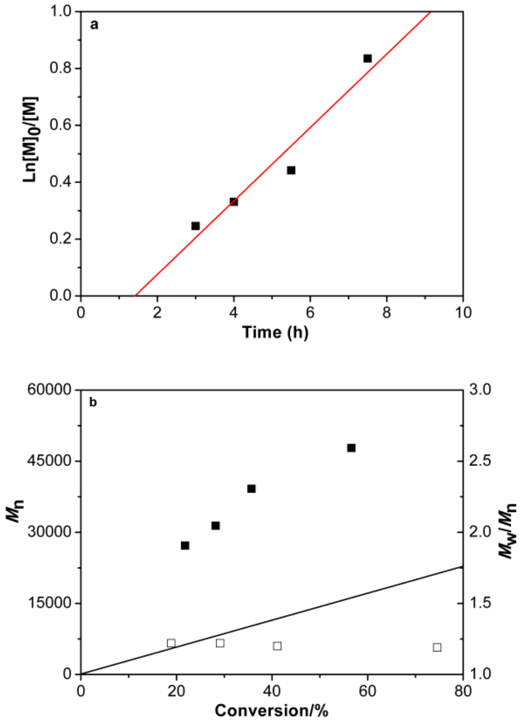

Figure S2. First-order plots (a) and evolution of M_n and $Đ$ with conversion (b) for the bulk $FeBr_2$-catalyzed MMA polymerization in the presence of PEO_{2000}-Br. [MMA]/[$FeBr_2$]/[PEO_{2000}-Br] = 200:0.5:1, T = 60 °C.

a. Conversion = 21.8%

Workbook Details

Eluent: THF

Flow Rate: 1.00 ml/min

Column Set:

Column Set Length: 0 mm

Detector: RI

Temperature: 30

Analysis Using Method: RI201702160

Comments:

Results File: E:\Cirrus Workbooks\2017\RI20170216\imported-0277.rst

Calibration Used: 2017/2/16 15:26:20

Calibration Type: Narrow Standard

Curve Fit Used: 1

Calibration Curve: y = 12.148291 - 0.515182x^1

High Limit MW RT: 11.03 mins

Low Limit MW RT: 18.27 mins

High Limit MW: 2911490

Low Limit MW: 546

K: 14.1000

FRM Name:

Alpha: 0.7000

Flow Marker RT: 0.00 mins

FRCF: 1.0000

MW Averages

Mp: 36857	Mn: 28364	Mv: 33514	Mw: 34361
Mz: 39932	Mz+1: 45004	PD: 1.2114	

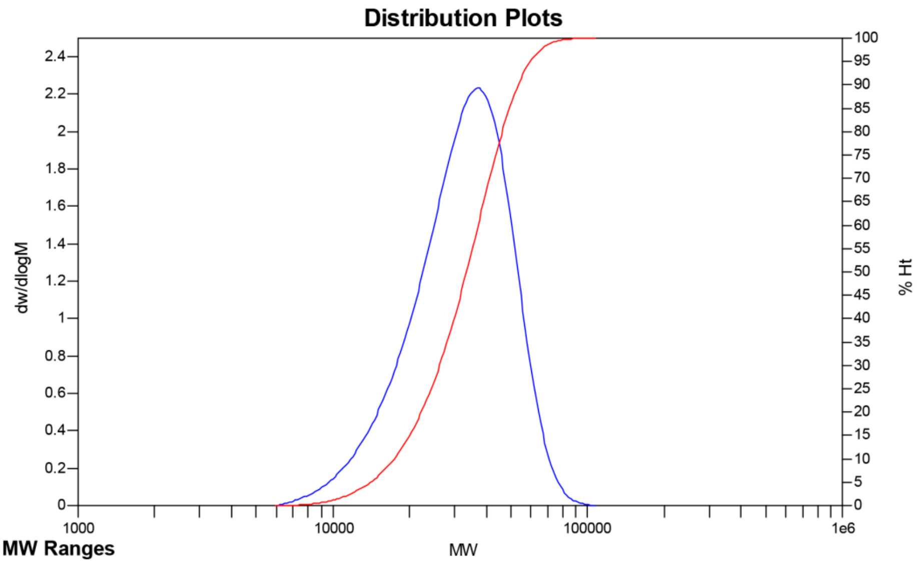

MW Ranges

b. Conversion = 28.2%

Workbook Details

Eluent: THF

Flow Rate: 1.00 ml/min

Column Set:

Column Set Length: 0 mm

Detector: RI

Temperature: 30

Analysis Using Method: RI201702160

Comments:

Results File: E:\Cirrus Workbooks\2017\RI20170216\imported-0278.rst

Calibration Used: 2017/2/16 15:26:20

Calibration Type: Narrow Standard

Curve Fit Used: 1

Calibration Curve: y = 12.148291 - 0.515182x^1

High Limit MW RT: 11.03 mins

Low Limit MW RT: 18.27 mins

High Limit MW: 2911490

Low Limit MW: 546

K: 14.1000

FRM Name:

Alpha: 0.7000

Flow Marker RT: 0.00 mins

FRCF: 1.0000

MW Averages

Mp: 44035	Mn: 32431	Mv: 38702	Mw: 39699
Mz: 46152	Mz+1: 51847	PD: 1.2241	

Distribution Plots

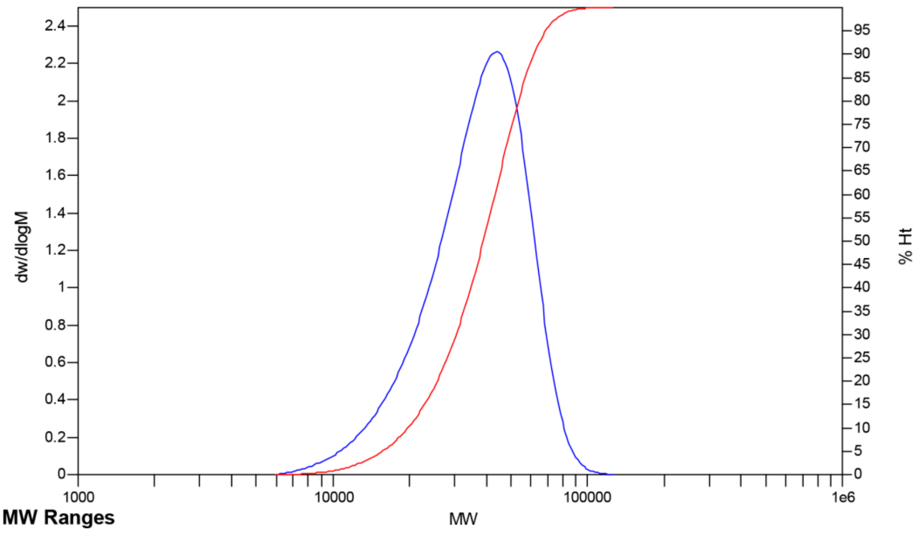

MW Ranges

c. Conversion = 35.7%

Workbook Details

Eluent: THF

Flow Rate: 1.00 ml/min

Column Set:

Column Set Length: 0 mm

Detector: RI

Temperature: 30

Analysis Using Method: RI201702160

Comments:

Results File: E:\Cirrus Workbooks\2017\RI20170216\imported-0279.rst

Calibration Used: 2017/2/16 15:26:20

Calibration Type: Narrow Standard

Curve Fit Used: 1

Calibration Curve: y = 12.148291 - 0.515182x^1

High Limit MW RT: 11.03 mins

Low Limit MW RT: 18.27 mins

High Limit MW: 2911490

Low Limit MW: 546

K: 14.1000

FRM Name:

Alpha: 0.7000

Flow Marker RT: 0.00 mins

FRCF: 1.0000

MW Averages

Mp: 53662	Mn: 40653	Mv: 47391	Mw: 48459
Mz: 55324	Mz+1: 61360	PD: 1.1920	

Distribution Plots

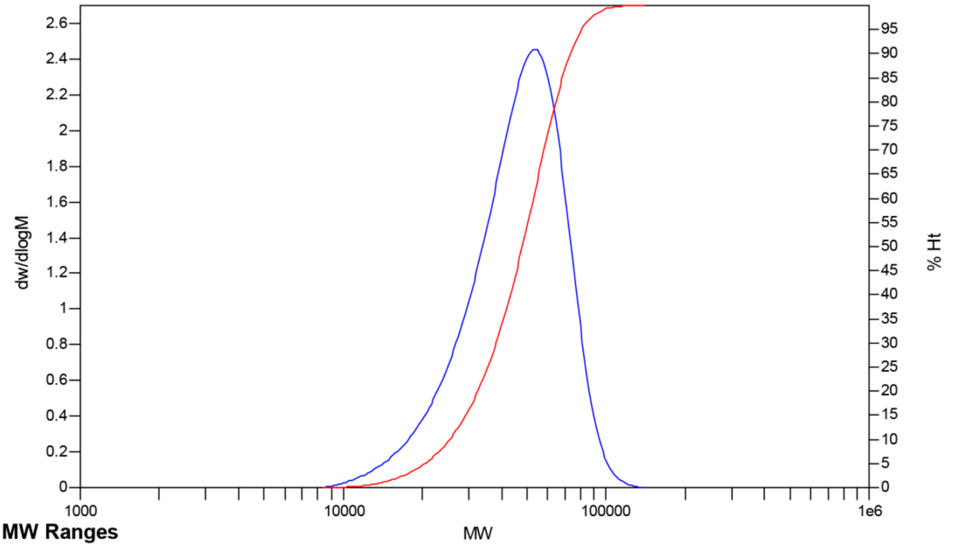

MW Ranges

d. Conversion = 56.6%

Workbook Details

Eluent: THF

Flow Rate: 1.00 ml/min

Column Set:

Column Set Length: 0 mm

Detector: RI

Temperature: 30

Analysis Using Method: RI201702160

Comments:

Results File: E:\Cirrus Workbooks\2017\RI20170216\imported-0280.rst

Calibration Used: 2017/2/16 15:26:20

Calibration Type: Narrow Standard

Curve Fit Used: 1

Calibration Curve: y = 12.148291 - 0.515182x^1

High Limit MW RT: 11.03 mins

Low Limit MW RT: 18.27 mins

High Limit MW: 2911490

Low Limit MW: 546

K: 14.1000

FRM Name:

Alpha: 0.7000

Flow Marker RT: 0.00 mins

FRCF: 1.0000

MW Averages

Mp: 65393	Mn: 49378	Mv: 57133	Mw: 58341
Mz: 66036	Mz+1: 72690	PD: 1.1815	

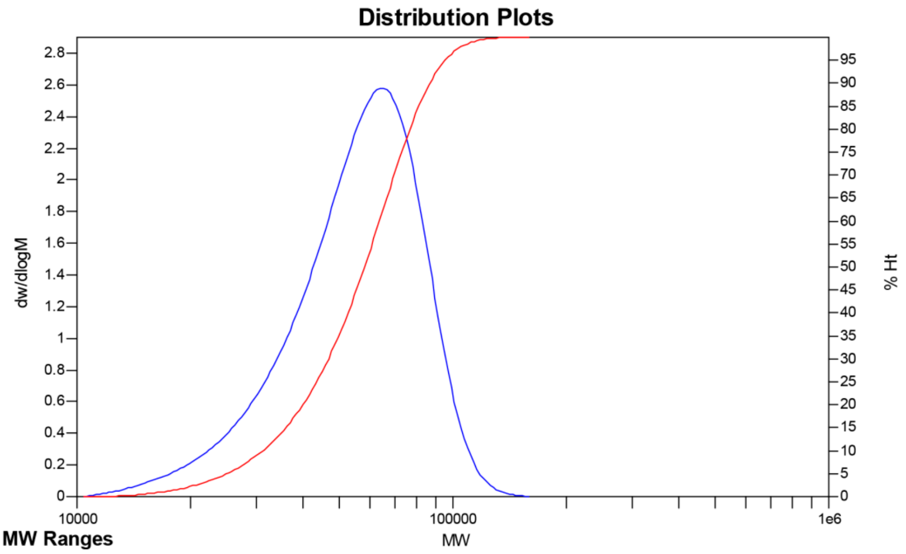

MW Ranges

Figure S3. Original GPC data with different monomer conversion related to Figure S1: a. 21.8%; b. 28.2%; c. 35.7%; d. 56.6%.

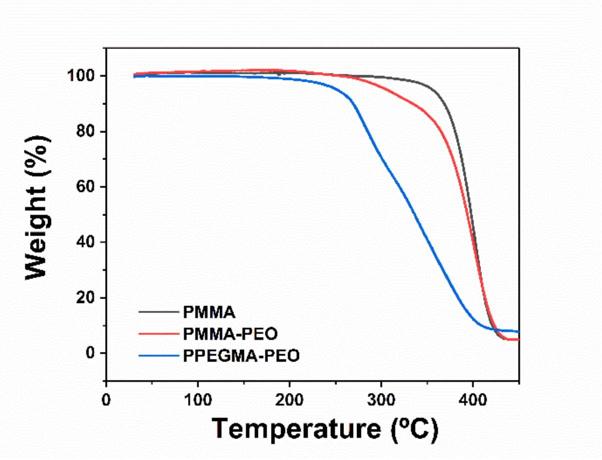

Figure S4. TGA of PMMA, PMMA-PEO2000 and PPEGMA-PEO2000.

a.

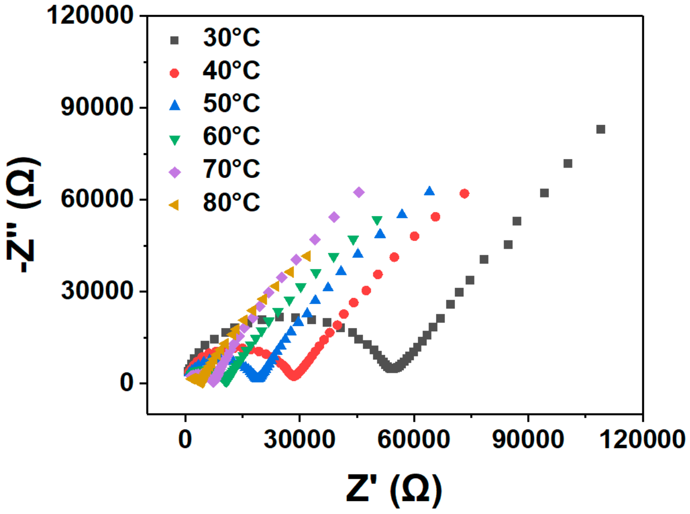

b.

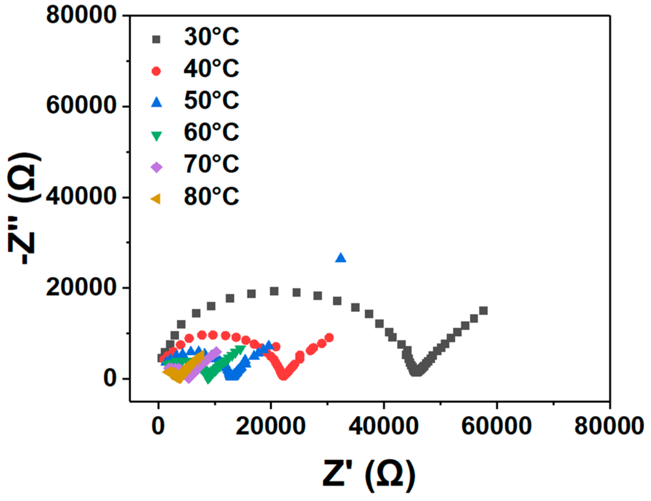

c.

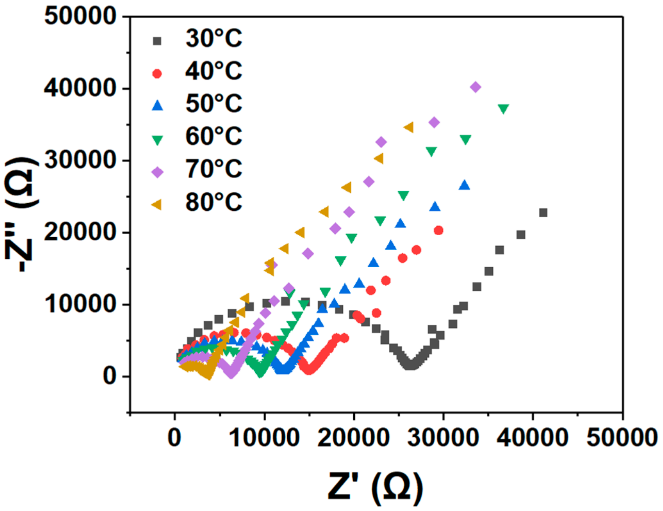

Figure S5. Original ac impedance spectra for calculating the ionic conductivity in Figure 5. a. M_n = 22300 g mol^{-1}; b. M_n = 38000 g mol^{-1}; c. M_n = 61700 g mol^{-1}.
